# The Treatment of Œdema in Chronic Nephritis

**Published:** 1911-04-29

**Authors:** F. W. W. Griffin

**Affiliations:** The Children's Infirmary, Carlshalton, Surrey.


### The General Practitioner's Column.

[Contributions to this Column are invited, and if accepted, will be paid for.]

### THE TREATMENT OF ŒDEMA IN CHRONIC NEPHRITIS.

By F. W. W. GRIFFIN, M.A., M.B., B.C., M.R.C.S., L.R.C.P., The Children's Infirmary, Carlshalton, Surrey.

The evil of being tied down to a fixed routine of treatment in chronic nephritis would scarcely need to be mentioned if it were not so common and so injurious. For instance, the principle of attempting to flush out chronically inflamed kidneys may be carried so far as to jeopardise gravely the patient's life. The occurrence of œdema would seem to indicate that more fluid is being taken than the kidneys can excrete, and should therefore serve as a warning to reduce the amount of fluids taken. If this reduction is not made, the accumulation of fluid in the tissues and peritoneal cavity increases, and the strain of work thus thrown upon the kidneys and heart becomes steadily greater until a fatal result may occur. On the contrary, if this warning is heeded, and the supply of fluid is sternly reduced until it becomes and remains less than the amount of fluid excreted, the pressure of work is lessened, œdema decreases, and some degree of compensation is obtained.

The first patient, a boy aged fourteen, had been under treatment for chronic parenchymatous nephritis without œdema for some months. Œdema supervened, and was not relieved by free diaphoresis and purgation, but rather increased, and fluid collected in the abdominal cavity. Reduction of the fluids taken was effected, and the amount given in the twenty-four hours was not allowed to exceed 15 ounces, then the limit of the excretory ability of the kidneys. On the fourth day after the commencement of the treatment there was noted an increase in the amount of urine passed, and a still further increase on the sixth day, from which date no marked diminution occurred, the œdema and ascites rapidly disappeared, and the general condition steadily improved. No diuretic drugs were employed until the improvement was definitely established. The second case is even more instructive. The patient was a boy, aged eleven, suffering from chronic parenchymatous nephritis with a considerable amount of ascites, palpable enlargement of both kidneys, and much œdema of the legs, back and face, with almost constant vomiting necessitating rectal feeding. This acute condition improved at first when the fluids taken were reduced, and there was an increase in the amount of urine passed. A diuretic mixture, with freer allowance of fluids, was then tried, with the result that the ascites and œdema increased again. The fluids were then definitely decreased to as low a limit as possible, which was maintained without reference to the amount of urine passed. This latter increased at first, and then became stationary at about 20 ounces in the twenty-four hours, the œdema decreasing more slowly than in the former case. There then occurred an acute attack, apparently of an uræmic nature, with repeated attacks of vomiting and severe suprapubic pain, the quantity of urine passed being diminished by about one-half. An increase of fluid was allowed, and free sweating and purgation induced.

Improvement quickly occurred and the fluids taken were again reduced, but this time only until they reached a level just below the amount excreted; as this amount increased, which it continued to do, the amount of fluid allowed was proportionately increased. The œdema and ascites diminished more rapidly now, and in the end disappeared completely, the subsequent progress being uninterrupted.

Whilst it is quite inadmissible to dogmatise on scanty evidence, the above two cases certainly suggest that a carefully watched, but not excessive, reduction of fluids taken may be a valuable part of the treatment of chronic nephritis.

